# Experimental study on rheological properties of serrate discontinuity under stepwise loading

**DOI:** 10.1038/s41598-025-04492-5

**Published:** 2025-07-01

**Authors:** Guang-hui Tian, Bao-qing Mei, Qing-zhao Zhang, Yu-xi Hao, Yan-zhi Zhu, Yong-zhi Jiu

**Affiliations:** 1 The School of Intelligent Construction and Civil Engineering, Zhongyuan University of Technology, No. 41 Zhongyuan Road (M), Zhengzhou, 450007 Henan Province China; 2 Department of Geotechnical Engineering, Tongji University, Shanghai, 200092 China

**Keywords:** Serrate discontinuity, Creep, Stress relaxation, Long-term strength, Civil engineering, Mechanical properties

## Abstract

Serrate discontinuity creep and stress relaxation tests are conducted to analyze the differences and relationships between creep, relaxation, and long-term strength using the same normal stress and shear stress levels. Both the creep and relaxation curves may be classified into two different phases: decelerating and steady. However, the trend of the creep curve is opposite to that of the stress relaxation curve. Additionally, we observed that the maximum creep displacement and maximum relaxation stress increase as the slope angle increases. The energy changing properties of rocks during creep and relaxation processes are unequal, as indicated by experiment results, indicating that creep and relaxation are not equal. An empirical model has been developed for the maximal relaxation stress and the shear stress level, which provides a better fit to the experimental data. Based on the same mechanism, the long-term strength determined by the creep test and the relaxation test shows little difference and is both close to the yield strength of the stress-displacement curves during the creep and relaxation processes, respectively. This study can provide a deeper understanding of the time-dependent characteristics of discontinuities by integrating creep, relaxation, and long-term strength.

## Introduction

Long term response and stability of rock masses engineering structures such as tunnels, underground openings and slopes have been receiving great attention since early times. Engineering practice shows that the stress and strain of rock mass will change and evolve over time, and even lead to rock mass destruction in the end. Rock masses possess significant time-dependent properties, known as rheological properties, that involve two aspects: creep, which causes gradual increase in strain with time under constant stress, and stress relaxation, which causes gradual decrease in stress with time under constant stress. Considerable study has been conducted to establish laws for variations in stress and strain over time, prompted by creep and relaxation. Many researchers performed creep test on different types of rocks such as Ito and Sasajima^1^, Brijes, et al.^2^ andHuang et al.^3^. Other scholars, meanwhile, conducted uniaxial and triaxial stress relaxation tests on rocks that are still intact, such as Ladanyi and Johnston^4^, Tian et al.^5^ and Paraskevopoulou et al.^6^.

Rock mass is a complex geological body that has many discontinuities, including fractures, weak bedding, and joints. These discontinuities give rise to the heterogeneity, anisotropy, and discontinuities of rock masses, which in practical engineering can even control the creep and relaxation characteristics of rock masses^7^. There is a big difference in creep and relaxation properties between intact rocks and rock masses because of the existence of the discontinuity in the actual project. Therefore, many scholars have studied the creep and relaxation characteristics of the discontinuity. Asanov et al.^8^ conducted shear creep testing on salt rock joints, and found that a typical shear creep curve of joints included three phases: a transient phase, a stable phase, and an accelerating phase, which corresponds to the process of traditional compressive creep tests. Wang et al.^9^ performed a series of stepwise loading creep tests on artificial rock discontinuities, and proposed a method for predicting the accelerating phase. Zhang et al.^10^ conducted creep tests on regular dentate discontinuity and found that steady creep does not exist in the strict sense. The so-called steady creep is in fact the approximately steady process of the creep velocity decreasing with time. Despite the extensive amount of compressive shear creep tests performed in the past decades, shear relaxation tests have received less research due to the potential complexity of relaxation test equipment. Liu et al.^11^ constructed artificial joint surfaces using cement mortar, evaluated them for shear stress relaxation, and developed a technique to calculate the joint’s long-term strength under cyclic loading. Wang et al.^12^ tested artificial and natural discontinuities with iso-stress cyclic and shear relaxation, and examined the relaxation behavior and how stress history and surface morphology affect stress relaxation in rock discontinuities. Tian et al.^13,14^ tested serrate discontinuities with various slope angles by employing shear creep and shear relaxation. They investigated the shear creep and shear relaxation characteristics of discontinuities when subjected to different loading paths, and came up with a data analysis technique for relaxation tests using stepwise loading. The above studies indicate that the shear creep and relaxation properties of serrate discontinuities are closely related to the joint roughness, normal load, and material strength, and so on.

Liu^15^ considered creep and relaxation to be equivalent in essence. There are only idealized mechanical concepts of materials’ long-term mechanical properties, which are fundamentally governed by an identical physical mechanics mechanism. There is a close inner link between creep and relaxation. At present, the investigation of the creep and relaxation characteristics of rock mass is being conducted according to their respective characteristics. It is quite necessary to make a comprehensive comparative analysis of creep and relaxation characteristics of rock mass, so as to gain a better understanding of the mechanical characteristics of rock masses that are subjected to long-term loads, and further understand the difference and internal relationship between creep and relaxation.

In order to reduce the discreteness of test results and the complexity of discontinuity, the creep characteristics and stress relaxation characteristics of discontinuity were studied by using serrate discontinuity with different slope angles. Based on test results, the basic laws of creep characteristics and relaxation characteristics of discontinuity with different slope angles, as well as the similarities and differences between the basic laws of creep and relaxation are analyzed. Simultaneous, the whole process stress-displacement curve of creep and relaxation are compared and analyzed. Besides, the long-term strength that is respectively determined by creep tests and relaxation tests, and is analyzed and compared with the yield strength of the stress-displacement curves during creep process and relaxation process. The combination of creep, relaxation and long-term strength in the paper can contribute to deeply understand the time-dependent property of discontinuity.

## Testing methodology

### Specimen

**Fig. 1:**
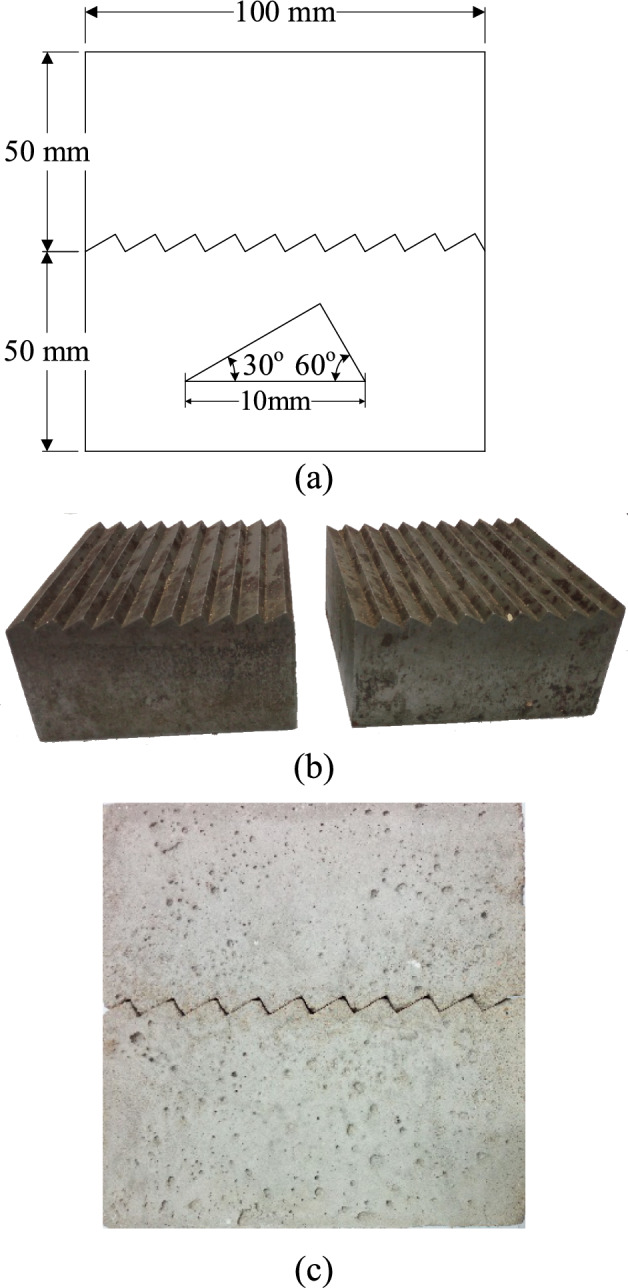
The specimen with a serrated surface: (**a**) Dimensions, (**b**) Section size of the 30° discontinuities, (**c**) joint molds.

The cement mortar sample, which is a rock-like material, is widely acknowledged and utilized to study the rheological properties of discontinuities with varying slope angles, and a significant amount of test data and research results have been obtained^11–13,16,17^. The sample with serrated surfaces measured 100 mm in length, 100 mm in breadth, and 100 mm in height. In the final sample, each tooth had a length of 10 mm, and there were 10 tooth shapes, as shown in Fig. 1(a). The regular cube sample is composed of two half specimens with different slope angles, as shown in Fig. 1(b). And there is a discontinuity in the middle of the sample, as depicted in Fig. 1(c). After 24 h, two half specimens were demoulded and cured with a constant temperature (20 ± 2 °C) and humidity (RH > 95%) for 28 days. 20 ± 2 °C and RH > 95%

Water, cement, and standard sand were mixed together in a ratio of 1:2:4. The cement is Portland blast furnace slag cement grade 32.5R according to the GB 175-2007 standard of China. This study’s test variable was the wedge slopes, which had values of 10°, 30° and 45°.

### Test equipment

**Fig. 2:**
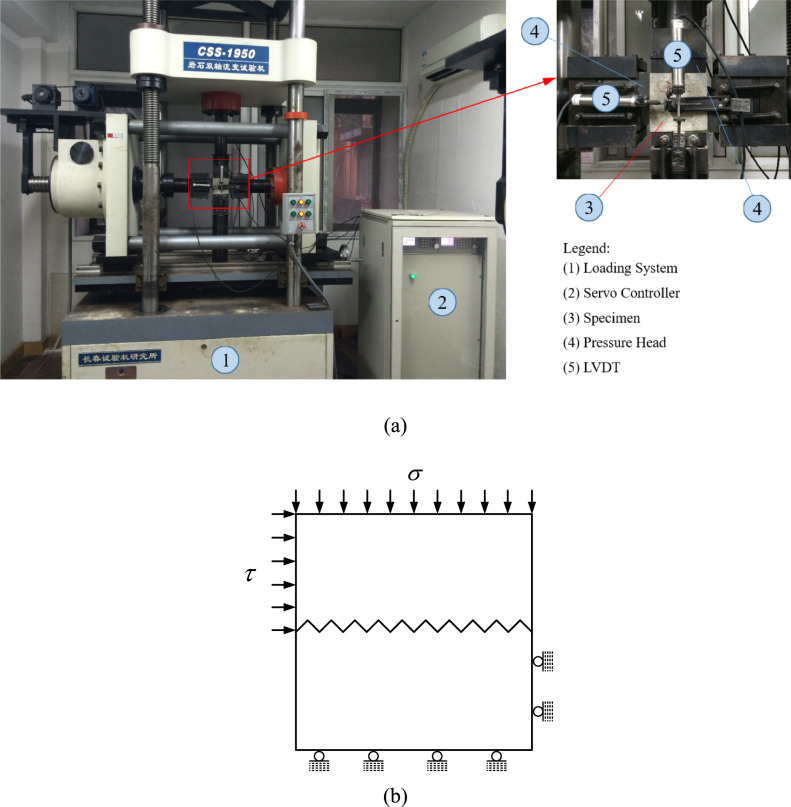
Test Equipment and Schematic diagram of loading direction: (**a**) Test Equipment, (**b**) Schematic diagram of loading direction.

Direct shear tests, shear creep tests, and shear stress relaxation tests were conducted on a rheological testing machine (CSS-1950. Figure 2) that was controlled by a servo. The maximum capacity in the vertical and horizontal directions of testing machine is 500kN and 300kN, respectively. During testing, also both compressive normal load and shear load can be simultaneously applied to the specimen, and the shear displacements of samples were measured using displacement transducers (LVDTs), with a range of 6 mm and an accuracy of 0.001 mm, respectively. The loading direction for the direct shear tests, creep tests and stress relaxation tests are shown in Fig. 2(b).

### Loading method

Three complete cube specimens without discontinuity were subjected to uniaxial compression tests, which revealed an average compressive strength *σ*_c_ of 19.62 MPa. Then, 1.962 MPa, 3.924 MPa, and 5.886 MPa, which is 10%, 20%, and 30% of the average uniaxial compressive strength of the complete cube specimen, were chosen as normal stresses for direct shear testing. In addition, three discontinuity specimens (slope angles of 10°, 30° and 45°) were selected for direct shear tests in order to determine the shear strength. Table 1 depicts the average shear strength of discontinuities for various slope angles under various normal loads.

**Table 1:** Average shear strengths of discontinuities.

Normal Stress /MPa	Slope Angle /°	Shear Strength *τ*_max_/MPa
1.962	10	1.69
1.962	30	2.60
1.962	45	3.57
3.924	10	3.40
3.924	30	4.40
3.924	45	5.59
5.886	10	4.82
5.886	30	5.93
5.886	45	7.58

Nowadays, the creep test method is the main basis for studies on the stress relaxation of rock masses. For example, Yu et al.^18^ and Paraskevopoulou et al.^6^ employed the stepwise loading method to perform uniaxial and triaxial relaxation experiments on rock. Additionally, the stepwise loading method is utilized to examine the relaxation property of discontinuities^12,14^. Consequently, the creep and relaxation tests in this study are conducted using the stepwise loading method. The shear stresses used in the stepwise loading experiments were 40%, 60%, 80%, 90%, and 95% of the discontinuity’s average shear strength at corresponding slope angles.

**Fig. 3:**
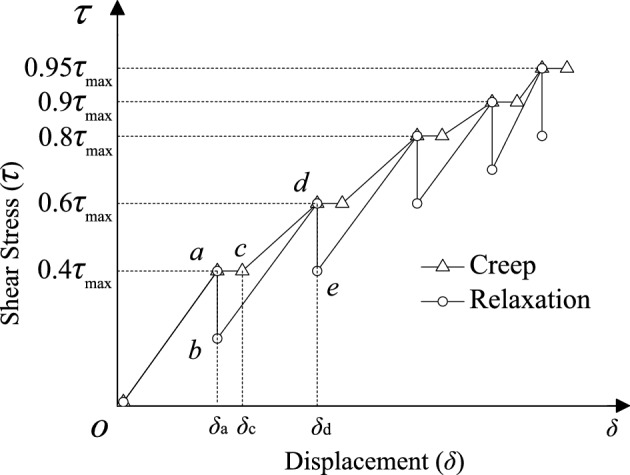
The loading paths for the creep and relaxation tests.

1. The creep test is illustrated in Fig. 3. The normal stress was applied at a rate of 0.02 MPa/s to 1.962 MPa and then kept constant. By 0.02 MPa/s, the shear stress increased and it reached 40% of the shear strength (i.e. from Point *o* to Point *a* in Fig. 3) after the normal displacement was stable. Afterwards, the shear stress remained at a constant level for 72 h, and during that time, the creep displacement increased from Point *a* to Point *c* in Fig. 3. After that the specified shear stress were applied at the same rate step by step, and each level of shear stress was maintained for 72 h.
2. The normal stress was applied at a rate of 0.02 MPa/s to 1.962 MPa and then kept constant. By 0.02 MPa/s, the shear stress increased and it reached 40% of the shear strength (i.e. from Point *o* to Point *a* in Fig. 3) after the normal displacement was stable. Afterwards, the displacement *δ*_a_ remained at a constant level for 72 h, and during that time, the shear stress relaxed from Point *a* to Point *b* in Fig. 3. After that the specified shear stress were applied at the same rate step by step, and the displacement generated at each level of shear stress was maintained for 72 h.

## Experimental results and discussion

### Characteristics of creep and relaxation for discontinuity

**Fig. 4:**
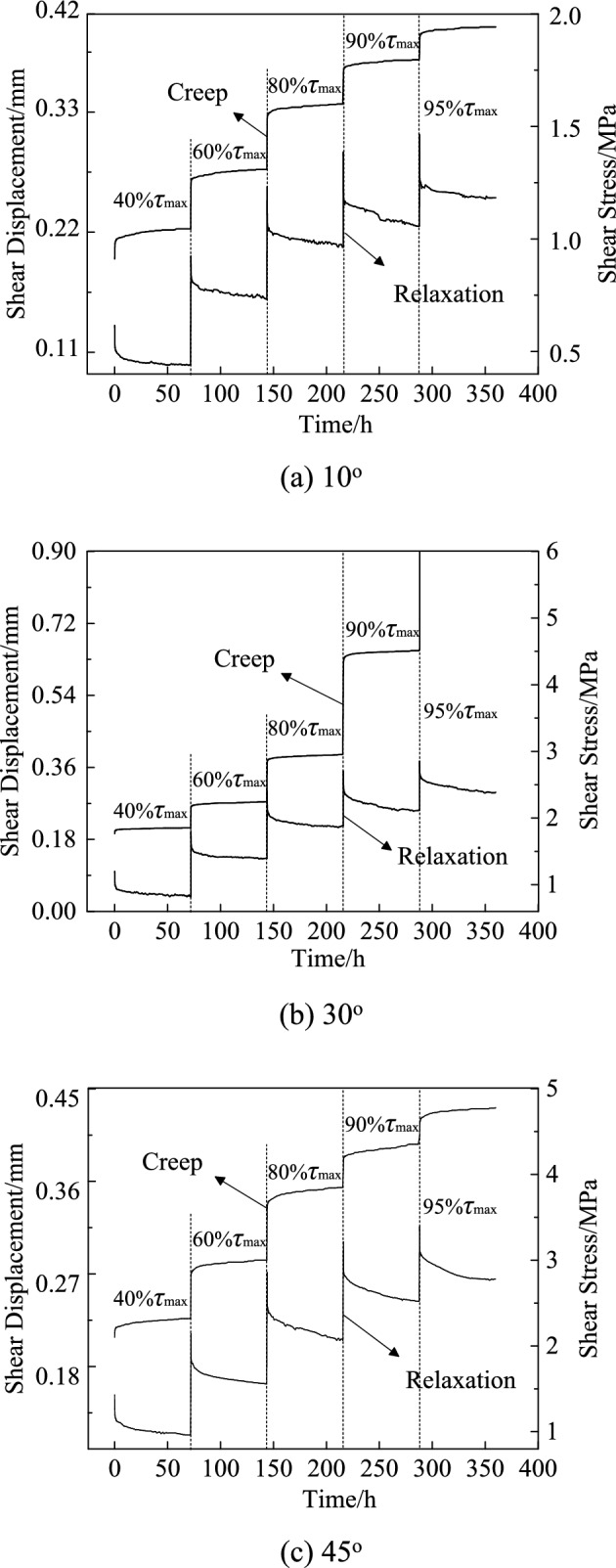
The whole process curve for creep and relaxation under normal stress of 1.962 MPa.

The whole creep and relaxation process curves for discontinuities with slope angles of 10°, 30° and 45° were obtained as seen in Fig. 4. Shear creep and relaxation have the following basic features.

1. During the creep, the shear displacement grew with time, whereas the displacement rate decreased gradually. The creep curve exhibits a convex appearance and is a continuous curve. As the shear stress level increases, the whole creep process curve takes on the shape of a ladder.

2. During relaxation, shear stress decreased with time, but does not reach zero. The relaxation curve is similarly a continuous curve, but has a concave shape and follows the opposite trend as the creep curve. As the level of shear stress increases, the whole relaxation process curve declines in a ladder-like manner.

3. According to Fig. 4b, at the 3th stage of loading (80%$\tau_{{{\text{max}}}}$), the instantaneous displacement for discontinuities with slope angles of 30° exhibits an anomalous rise, indicating that the sample has now reached yield. As a result, the sample suffers significant creep displacement in the 3th stage (80%$\tau_{{{\text{max}}}}$) of the shear stress level and is destroyed in the 4th stage (90%$\tau_{{{\text{max}}}}$), but it does not destroy in relaxation testing due to a difference in sample property.

4. The creep curve and relaxation curve are not smooth, with sudden rises and drops of the shear displacement (during creep test) and the shear stress (during relaxation test) occasionally. The rules we have observed relate to sample damage, as well as the choice between energy build-up and energy loss during creep and relaxation tests. Other scholars^19–21^ also observed similar characteristics.

### Effect of slope angle on creep and stress relaxation

**Fig. 5:**
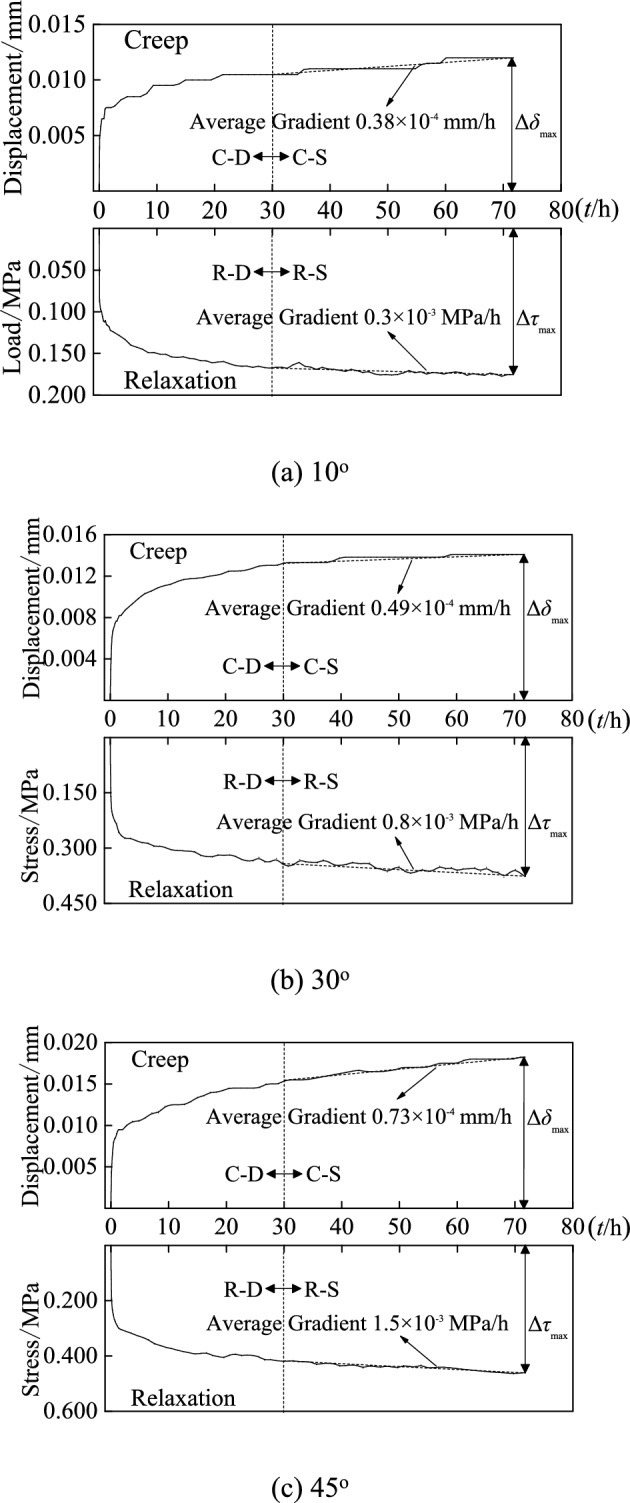
Creep and relaxation curves for discontinuities with varying slope angles during the 1st stage (C-D: the stage where creep decelerates; C-S: the stage where creep is steady; R-D: the stage where relaxation decelerates; R-S: the stage where relaxation is steady).

To prevent the effect of loading history on test data, Fig. 5 displays the creep and relaxation curves for three slope angles of 10°, 30° and 45° at the 1st stage (40%$\tau_{{{\text{max}}}}$) of the shear stress level, with a normal stress of 1.962 MPa. The creep curve and stress relaxation curve of discontinuities with various slope angles are plotted on the same time-scale, i.e., starting at zero, as seen in Fig. 5. In the meantime, the downward direction is interpreted as positive in the relaxation curve and the upward direction as positive in the creep curve.

Figure 5 illustrates the general properties of shear creep and relaxation:

1. The displacement/stress gradually increases/decreases, and both the creep and relaxation curves flatten out with time, indicating that both the creep and relaxation rate reduce over time, but the discontinuity’s stress does not relax to zero.
2. The creep and relaxation curves are demarcated by the rate of 30th hour. There are two stages in the creep curve: the decelerating stage (C-D) and the steady stage (C-S), and there are also two stages in the relaxation curve: the decelerating stage (R-D) and the steady stage (R-S). In the creep and relaxation test, the average gradients of the stable stage (stages C-S and R-S) increase as the slope angle increases. In stage C-S, the average rate of specimens with slope angles of 10°, 30° and 45° for creep during 30–72 h has an average rate of 0.38 × 10^−4^ mm/h, 0.49 × 10^−4^ mm/h and 0.73 × 10^−4^ mm/h, respectively. In stage R-S, the average rate of specimens with slope angles of 10°, 30° and 45° for relaxation during 30–72 h has an average rate of 0.3 × 10^–3^ MPa/h, 0.8 × 10^–3^ MPa/h and 1.5 × 10^–3^ MPa/h, respectively.
3. During creep test, the difference between initial displacement *δ*(0) at the beginning of the creep test (i.e., at *t* = 0) and the final displacement *δ*(72) after 72 h is defined as maximum creep displacement Δ*δ*_max_, i.e., Δ*δ*_max_ = *δ*(72)-*δ*(0), as shown in Fig. 5. During relaxation test, the difference between initial shear stress $\tau (0)$ (i.e., at time *t* = 0) and the residual shear stress $\tau (72)$ after 72 h is determined as the maximum relaxation stress $\Delta \tau_{\max }$, i.e., $\Delta \tau_{\max } = \tau (0) - \tau (72)$, as depicted in Fig. 5. In addition, the maximum creep displacement Δ*δ*_max_ and maximum relaxation stress $\Delta \tau_{\max }$ are found to increase as slope angles rise, implying that slope angle greatly impacts on the creep and relaxation properties of discontinuities.

### The stress-displacement curve during creep and relaxation

**Fig. 6:**
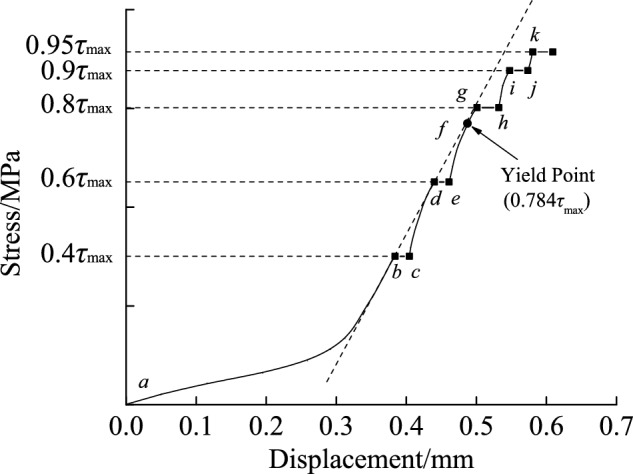
The stress-displacement curve for creep with a normal stress of 1.962 MPa.

**Fig. 7:**
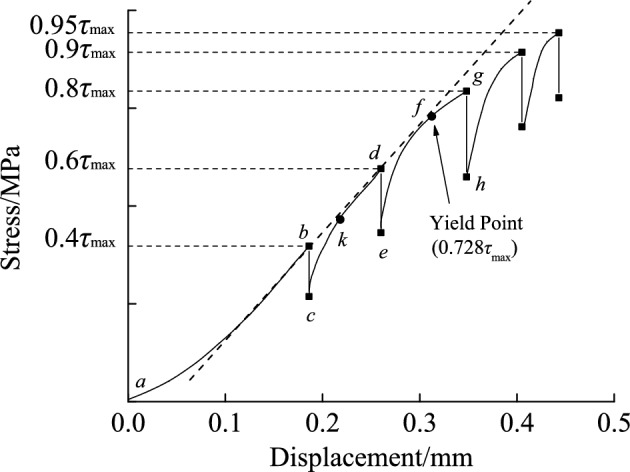
The stress-displacement curve during relaxation with a normal stress of 1.962 MPa.

To examine the similarities and differences between creep and relaxation, consider a discontinuity with slope angle of 45°, with a normal stress of 1.962 MPa. The stress-displacement curves for the whole creep and relaxation phase are shown in Figs. 6 and 7, respectively.

## It can be seen from the curves in Fig. 6 that:

As a result of the sample’s two structural surfaces not being in close touch and microcracks opening, the sample was first compacted before entering the elastic segment during the first stage of loading (path *a* → *b* in Fig. 6), resulting in a concave curve. After creep in the first stage (path *b* → *c* in Fig. 6), the two structure surfaces contacted closely, which caused microcracks to be squeezed and gradually closed, resulting in a hardening phenomenon in the sample. Fan et al.^22^ and Fan et al.^23^ both pointed out that the creep process involves continuously closing micropores and compressing the weak phase, which results in the improvement of the deformable structure’s strength and a hardening phenomenon in the sample. Consequently, during the loading process of the 2nd and 3rd stage (paths *c* → *d* and *e* → *g* in Fig. 6), the microfissure of the sample expanded to a certain extent, but the hardening effect remained dominant in creep, and the displacement of the sample approximately presented a linear variation with load. Due to the memory effect of rock displacement, the displacement curve advanced along the extension line direction of the elastic segment (similar to the dotted line in the diagram) in the loading curve after creep. When the sample entered the plastic yield region during the 4rth and 5th stages (path *h* → *i* and* j* → *k* in Fig. 6), the stress-displacement curve began to move toward the *X* axis, rather than following the initial monotonous loading curve. During the 3rd stage loading (i.e., path e → g in Fig. 6), the sample yielded with a stress of approximately 2.8 MPa and a ratio of 0.784 to shear strength.

## It can be seen from the curves in Fig. 7 that:

The characteristics of the stress-displacement curve during the relaxation test’s 1st stage loading (path *a* → *b* in Fig. 7) were identical to those of the creep tests. Tang et al.^24^ stated that elastic deformation energy stored in rock during loading would create new fissures in the rock and be released during the sliding process of the fracture surface. As soon as the displacement caused by the first stage loading process was constant, the relaxation process began. The elastic deformation energy accumulated during loading could cause a fissure in the medium that lacks sufficient strength in the specimen, or expand the microfracture, leading to significant dissipation of energy. When the remaining elastic deformation energy was insufficient to generate new fissures, the sample would adopt internal structural adjustment mode, such as fracture surface sliding, to consume energy until the internal and external loads were balanced. Internal sample structural adjustments, such as sliding and extruding of fractures, may also cause a degree of hardening effect during the relaxation process, comparable to the creep hardening phenomena. In Fig. 7, the stress-displacement curve of the loading section with all stages was clearly convex (i.e., path *c* → *d* and* e* → *g* ), indicating that the sample caused a hardening effect and increased tangent modulus at the reloading stress section. When the stress (point *k*) during the reloading process exceeded the initial stress (point *b*) of relaxation in the first stage, the hardening phenomenon and the memory effect of the rock displacement disappeared, and the displacement curve once again advanced along the extension line direction of the elastic segment (similar to the dotted line in the diagram). And the curve resembled the typical stress–strain curve of all relaxation stages obtained by Haupt^25^. Sample variations resulted in the sample yielding during the loading process of the third stage (path *e* → *g* in Fig. 7) in the relaxation test (the stress at the yield point was about 2.6 MPa, equivalent to 0.728 of the average shear strength), which differed slightly from the yield strength in the creep test.

## Long-term strength

### Determination of long-term strength by creep

The maximum load a rock can withstand while maintaining long-term stability is called long-term strength, which is often calculated indirectly by using creep testing. Among indirect methods, the steady-state creep rate method is a popular and simple approach. The mechanism for determining long-term strength through the steady-state creep rate method indicates that when the applied external loading is less than or equal to the long-term strength, the rate of stable creep stage is zero, and when it is greater than the long-term strength, the steady-state creep rate in the rock is non-zero^26^. Through shear creep testing, Zhang et al.^10^ discovered that the steady-state creep rate of regular dentate discontinuities is not constant but rather gradually changes over time under both low and high stress levels. This stage is generally considered to be steady creep due to the extremely slow creep rate. In other words, the creep rate during the steady creep stage is not exactly zero, but rather a constant close to zero. Consequently, long-term strength can be determined by using the inflection point of a sudden shift in the steady-state creep rate. Furthermore, Liu et al.^27^ and Cheng et al.^28^ argued that the inflection point of the steady-state creep rate reflects the long-term strength of the rock.

**Fig. 8:**
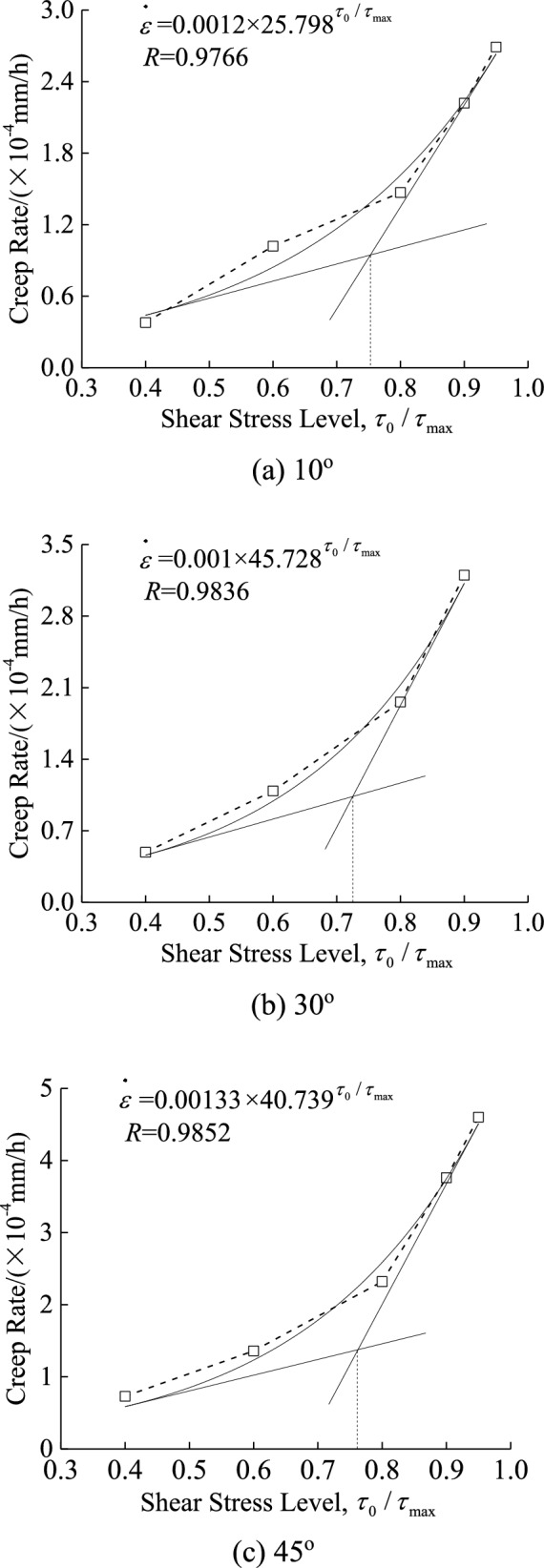
Relationship between steady-state creep rate and shear stress level.

Figures 4 and 5 show that after 24 h, the samples with different slope angles reached the steady-state creep stage. The average creep rate within the range of 30–72 h was chosen as the steady-state creep rate. The creep curve of different stress levels under single stage loading can be obtained based on the superposition principle from stepwise loading creep tests, and the relationship of steady-state creep rate with stress level for discontinuity with slope angle of 10°, 30°, and 45° is shown in Fig. 8. Because of the difference in the shear strength of discontinuity with varying slop angle, the applied initial shear stress varies as well. To facilitate comparison analysis, the horizontal axis in Fig. 8 adopts the ratio of initial shear stress to shear strength, i.e., $\tau_{0} /\tau_{\max }$.

As shown by Cheng et al.^28^ and Wu et al.^26^, the correlation between the steady-state creep rate and the shear stress level is well-represented by an exponential function. Therefore, the steady-state creep rate and the shear stress level $\tau_{0} /\tau_{\max }$ can be represented by an exponential function as follows

1 $$\dot{\varepsilon } = ab^{{\tau_{0} /\tau_{\max } }}$$

where $\tau_{0} /\tau_{\max }$ is shear stress level, which is the ratio of initial shear stress to shear strength, and *a* and *b* are material constants. Figure 8 displays the correlations between steady-state creep rate and shear stress level, and the fitting curves that correspond to slope angles of 10°, 30° and 45° under a normal stress of 1.962 MPa. A relatively good correlation coefficient was found. Since there is no clear inflection point for the exponential function, the paper utilized the intersection of the tangent of the beginning and end points of the fitted curve as the inflection point. This intersection marks the transition of the sample from steady creep to accelerated creep. Thus, the ratio of long-term strength $\tau_{\infty }$ to shear strength $\tau_{\max }$ was the shear stress level $\tau_{0} /\tau_{\max }$ that corresponded to the intersection.

According to the method described above, the shear stress level for discontinuities with slope angles of 10°, 30°, and 45° is determined by the intersection of the tangent, and is, thus, 0.753, 0.725, and 0.763, with an average value of 0.747. Among these, the value of $\tau_{0} /\tau_{\max }$ (i.e., 0.763) for the discontinuity with a 45° slope angle is close to the ratio (i.e., 0.784) between the yield strength and the shear strength in the stress-displacement curves during the creep test, as shown in Fig. 6. The average value of $\tau_{0} /\tau_{\max }$ for discontinuity with slope angles of 10°, 30° and 45°, 0.747, was taken as the stress level corresponding to the long-term strength. It was close to 0.8, which is the ratio of long-term strength to shear strength determined through isochronous curve when Hou et al.^29^ conducted creep tests on regular dentate discontinuity with slope angles of 30° and 45° under the same loading method.

### Determination of long-term strength by relaxation

#### Stress relaxation under different shear stress level

**Fig. 9:**
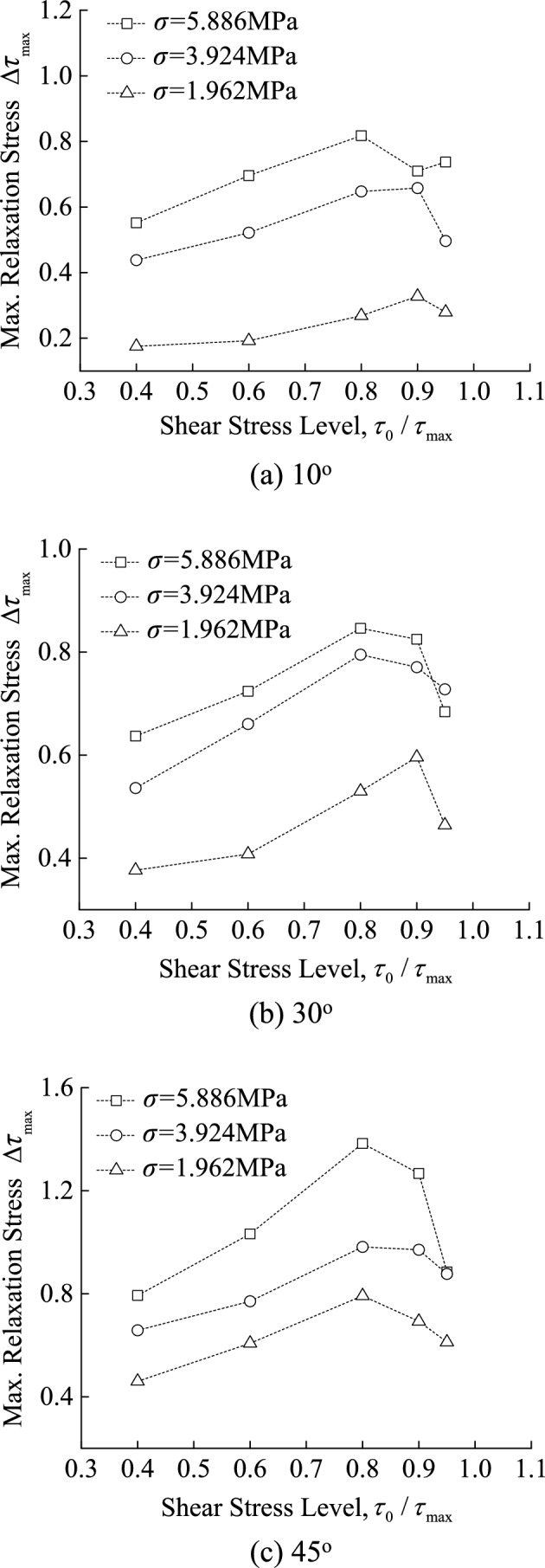
Relationship between maximum relaxation stress and shear stress level.

As previously stated, during the relaxation test, the difference between the initial shear stress $\tau (0)$ (i.e., at time *t* = 0) and the residual stress $\tau (72)$ after 72 h is defined as the maximum relaxation stress $\Delta \tau_{\max }$, i.e., $\Delta \tau_{\max } = \tau (0) - \tau (72)$. The relationship between the maximum relaxation stress $\Delta \tau_{\max }$ for various discontinuities and the shear stress level is depicted in Fig. 9. To facilitate comparison analysis, the horizontal axis also adopted the ratio of initial stress to shear strength, i.e., the value of $\tau_{0} /\tau_{\max }$.

It can be seen from the curves in Fig. 9 that:

1. With constant normal stress, the maximum relaxation stress $\Delta \tau_{\max }$ increase with slope angle at the same shear stress level ($\tau_{0} /\tau_{\max }$). For example, at the 1st shear stress level ($\tau_{0} /\tau_{\max }$ = 0.4), the maximum relaxation stress for the discontinuities with slope angles of 10°, 30° and 45° is 0.432 MPa, 0.536 MPa and 0.658 MPa, respectively, when the normal stress is 3.924 MPa.
2. With a constant slope angle of discontinuity, the maximum relaxation stress $\Delta \tau_{\max }$ increase with normal stress at the same shear stress level ($\tau_{0} /\tau_{\max }$). For example, at the 1st shear stress level ($\tau_{0} /\tau_{\max }$ = 0.4), the maximum relaxation stress is 0.377 MPa, 0.536 MPa and 0.637 MPa when the normal stress is 1.962 MPa, 3.924 MPa and 5.886 MPa for discontinuity with slope angles of 30°.
3. The maximum relaxation stress $\Delta \tau_{\max }$ increases with the shear stress level ($\tau_{0} /\tau_{\max }$), but there is a peak rather than an ongoing increase. For example, for the discontinuities with slope angles of 10°, the maximum relaxation stress peaks at shear stress level ($\tau_{0} /\tau_{\max }$) of 0.9 when the normal stress is 1.962 MPa and 3.924 MPa, and 0.8 when the normal stress is 5.886 MPa.
4. The shear stress levels corresponding to the peak of the maximum relaxation stress are listed in Table 2. According to the table above, the maximum relaxation stress occurs at the shear stress level ($\tau_{0} /\tau_{\max }$) of 0.9 when the slope angle and normal stress of the discontinuity are small. Nevertheless, when the normal stress is 5.886 MPa, the maximum relaxation stress occurs at the shear stress level ($\tau_{0} /\tau_{\max }$) of 0.8 for all discontinuities with different slope angles.

**Table 2:** The shear stress level $\tau_{0} /\tau_{\max }$ corresponding to the peak of $\Delta \tau_{\max }$.

Normal stress/MPa	Slope angles/°		
	10	30	45
1.962	0.9	0.9	0.8
3.924	0.9	0.8	0.8
5.886	0.8	0.8	0.8

As demonstrated by previous research, the slope angle, normal stress, and shear stress level significantly influence the relaxation behavior of discontinuities. There are two main types of shear displacement modes for serrate discontinuity: surface sliding and asperity crushing, surface sliding is the failure mode of discontinuity when the slope angle and normal stress are low, otherwise it is asperity crushing. Previous reports have documented similar findings^19,30,31^. As is well known, stress relaxation is the process of elastic displacement transforming into inelastic displacement^11,32,33^. Since surface sliding is the discontinuity failure mode when the slope angle and normal stress are low and consumes energy accumulated in the specimen during loading, a small amount of elastic energy generated in the specimen during loading results in less damage and a higher shear stress level $\tau_{0} /\tau_{\max }$ corresponds to the maximum relaxation stress $\Delta \tau_{\max }$. Asperity crushing is the failure mode that occurs when the slope angle and normal stress are high and a large quantity of elastic energy is generated. This causes greater specimen damage and and a lower shear stress level $\tau_{0} /\tau_{\max }$ corresponds to the maximum relaxation stress $\Delta \tau_{\max }$.

#### Long‑term strength of discontinuities

**Fig. 10:**
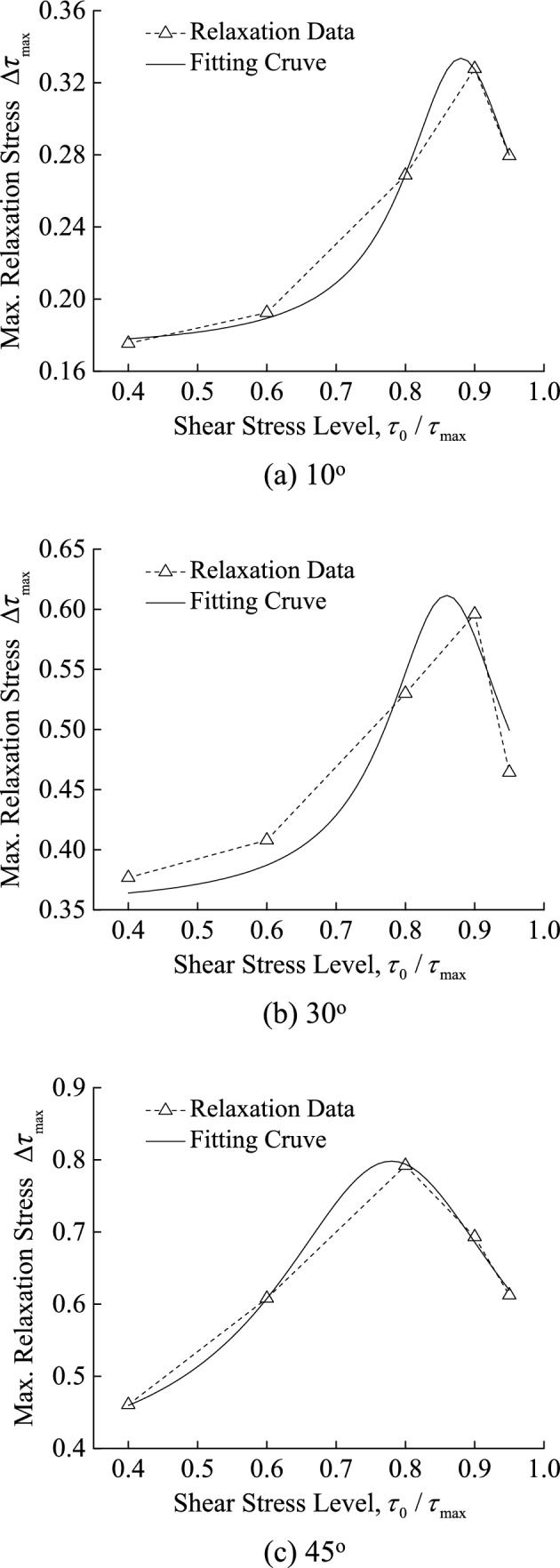
The fit curve between $\Delta \tau_{\max }$ and $\tau_{0} /\tau_{\max }$ under normal stress of 1.962 MPa.

According to the regression analysis of test data in Fig. 9, Eq. (2) may well characterize the maximum relaxation stress with shear stress level, i.e.,

2 $$y = y_{0} + A\frac{b}{{4(x - c)^{2} + b^{2} }}$$

where $y{}_{0}$, *A*, *b* and *c* are material constants. It can be known from the equation that the curve reaches the peak value $y_{0} + \frac{A}{b}$ when $x = c$. Figure 10 depicts the relationships between the maximum relaxation stress $\Delta \tau_{\max }$ and the shear stress level $\tau_{0} /\tau_{\max }$, as well as their corresponding fitting curves for slope angles of 10°, 30° and 45° under normal stress of 1.962 MPa, where the values of $y{}_{0}$, *A*, *b* and *c* in Eq. (2) are listed in Table 3. It can be seen from the curves in Fig. 10 and Table 3 that the Eq. (2) can match the maximum relaxation stress $\Delta \tau_{\max }$ with the shear stress level $\tau_{0} /\tau_{\max }$ but with different coefficient values, $y{}_{0}$, *A*, *b* and *c*.

**Table 3:** Regression Coefficients of Eq. (2).

Normal Stress /MPa	Slope Angle /°	$y{}_{0}$	*A*	*b*	*c*	*R*
1.962	10	0.1714	0.0321	0.1981	0.880	0.9958
1.962	30	0.3514	0.0540	0.2075	0.860	0.9230
1.962	45	0.3616	0.1784	0.4085	0.780	0.9982

The stress corresponding to the maximum relaxation stress $\Delta \tau_{\max }$ is the long-term strength, and the approach is the same as that used to determine long-term strength through the steady-state creep rate method^17^. Therefore, the coefficient *c* corresponding to the peak of the fitted curve can be considered as the ratio of long-term strength $\tau_{\infty }$ to shear strength $\tau_{\max }$, with an average value of 0.840. It differs from the average value of $\tau_{0} /\tau_{\max }$ corresponding to the long-term strength determined according to the creep test, 0.747, but the difference is only around 0.093, which is not significant. The shear stress level $\tau_{0} /\tau_{\max }$ corresponding to the long-term strength for discontinuity with slope angle of 45° determined in this way is 0.78, which is close to 0.728, the ratio of yield point strength to average shear strength of the stress-displacement curve during the relaxation process for discontinuity with slope angle of 45°, as shown in Fig. 7.

Due to the irreversibility of work and the difference in stress-displacement curves between creep and relaxation tests, the energy changing characteristics of rock during the creep and relaxation processes must be unequal. This leads to a slight variation in the long-term strength determined in creep and relaxation tests. Despite slight differences in long-term strength determined by the creep and relaxation tests, they cannot be considered equivalent since the energy change in the sample during the creep and relaxation tests is different, as indicated by the above analysis.

## Conclusions

This research characterizes the shear creep and relaxation of discontinuities with varying slope angles under different normal and shear stresses. Subsequently, a comprehensive analysis of creep, relaxation, and long-term strength was performed. Although the creep curve and the stress relaxation curve share many similarities according to experimental results, their trends are diametrically opposed. The creep curve is convex, while the relaxation curve is concave. It has been found that the maximum creep displacement in creep tests and the maximum relaxation stress in relaxation tests both increase with the slope angle of discontinuity at the 1st stage of the shear stress level. By observing and analyzing the stress-displacement curves for the whole creep and relaxation phase, it has been found that the energy changing characteristics of rock during the creep and relaxation processes are unequal, and creep and relaxation cannot be considered equivalent due to the irreversibility of work. Furthermore, it was discovered that, based on the same mechanism, the long-term strength determined by the creep test and the relaxation test have little difference and are both close to the yield strength of the stress-displacement curves during the creep and relaxation processes. The experimental results and analysis reported in the study can help to understand the connection between creep, stress relaxation, and long-term strength in a more complete and detailed manner. These findings could be used as a foundation for engineering applications and theoretical studies.

## Data Availability

The data that support the findings of this study are available from the corresponding author upon reasonable request.
